# Editorial: Interspecies Interactions: Effects on Virulence and Antimicrobial Susceptibility of Bacterial and Fungal Pathogens

**DOI:** 10.3389/fmicb.2020.01922

**Published:** 2020-08-26

**Authors:** Giuseppantonio Maisetta, Giovanna Batoni

**Affiliations:** Department of Translational Research and New Technologies in Medicine and Surgery, University of Pisa, Pisa, Italy

**Keywords:** interspecies interactions, polymicrobial infections, virulence, biofilm, susceptibility

One of the most exciting achievements that microbiologists have pursued over the last decades is the recognition that microorganisms rarely live as single fluctuating entities but strictly interact with each other in complex communities known as biofilms. Studies on dental biofilms, intestinal communities, chronic wounds, or respiratory infections in patients with cystic fibrosis clearly demonstrate that community interactions greatly influence microbial survival and disease progression (Dalton et al., 2011; Caverly et al., 2015; Reynolds et al., 2015; Marsh and Zaura, 2017). These interactions range from synergism to competition and involve, among others, physical interactions, chemical signaling, and exchange of genetic information. There is no doubt that consideration of the social behavior of microorganisms can reveal emergent traits and mechanisms of pathogenicity that would be overlooked by studying bacteria in isolation. For instance, community establishment provides members with additional properties such as enhanced tolerance to antimicrobials, ability to evade host immune responses or to survive in harmful environments (Batoni et al., 2016).

This Research Topic gathers 11 articles from 71 authors exploring different aspects of species-to-species interactions. We believe that a deep understanding of the mechanisms at the basis of the ecological interactions among microbial species will provide the knowledge needed to translate novel interventions for the diagnosis, treatment, and prevention of poly-microbial infections into the clinic, and hope that this Research Topic may contribute to this purpose.

Certainly, the study of interspecies interactions strongly relies on the availability of suitable experimental models that enable the stable and long-term cultivation of poly-microbial communities (Røder et al., 2020). In this respect, the Topic includes three studies aimed at reproducing the features of specific body sites and at exploring the multiple interactions among commensals and pathogenic organisms, as well as antimicrobials. O'Brien and Welch described a new continuous-flow model for *in vitro* cultivation of mixed bacteria associated with cystic fibrosis airway infections, while Jordana-Lluch et al. developed and validated a simple 2D skin infection model for investigating commensals, pathogens and keratinocytes interactions. Finally, by employing an *in vitro* nasopharyngeal colonization model that mimics the conditions of the human nasopharynx including temperature, nutrient availability, aeration, and epithelial attachment, Bair and Campagnani studied the co-colonization dynamics of three main othopatogens: *Moraxella catarrhalis*, non-typable *Haemophilus influenzae* (NTHi), and *Streptococcus pneumoniae* and found that the presence of *M. catarrhalis* is essential for NTHi to survive the bactericidal effects of *S. pneumoniae*.

One of the most obvious translational aspect of interspecies interactions is the use of “friend” microorganisms, the so-called probiotics, to prevent or cure diseases caused by microorganisms endowed high pathogenic potential. Although traditionally used to restore intestinal flora after prolonged antibiotic therapy, probiotics have been considered as means to prevent/treat a variety of diseases during the last decade (Sales-Campos et al., 2019). In this Research Topic, two papers concerning the employment of candidate probiotics against oral pathogens are included. Moman et al. demonstrated that bacterial strains such as *Lactobacillus reuteri* and *Streptococcus salivarius* decrease the toxic effects of the periodontal pathogens *Porphyromonas gingivalis* and *Aggregatibacter actinomycetemcomitans* toward oral keratinocytes and in an *in vivo* model of *G. mellonella* larvae. Zhang et al. reported that *Lactobacillus plantarum* K41 is able to inhibit biofilm formation of the highly cariogenic species *Streptococcus mutans* and observed a significant reduction in the incidence and severity of dental caries in rats pretreated with this probiotic strain.

Kang et al. showed that competitive interactions among microorganisms can be exploited to protect plants by phytopathogens. They studied how the gene expression of the pathogenic fungus *Gaeumannomyces graminis* var. *tritici* was affected by *Bacillus velezensis*, an endophytic biocontrol bacterium exhibiting a broad antifungal spectrum against many phytopathogens.

The close relationship between interspecies interactions and susceptibility to antimicrobials is a rapidly expanding research area that is likely to provide new clues to face the worrisome and world- spreading problem of antimicrobial resistance (AMR) (Radlinski and Conlon, 2018). In this direction, Banerji et al. present a comprehensive and interesting review addressing the relation between microbial interactions and AMR from an ecological point of view. Highlighting that human, animal, and environmental systems are strictly interconnected, the Authors show that species interactions may play significant and sometimes multifaceted roles in determining the prevalence and distribution of AMR and antimicrobial resistance-associated genes (ARGs).

The effects of interspecies interactions on antibiotic susceptibility are not limited to closely related microorganisms but can actually cross kingdom borders (Harriott and Noverr, 2009). Nabb et al. disclose an interesting mechanism by which *C. albicans* may promote multi-drug tolerance in *S. aureus*. They report that *S. aureus* grown in dual cultures with *C. albicans* displays decreased intracellular ATP concentrations as well as lower membrane potential when compared to cultures lacking *C. albicans*. Collectively, the data reported demonstrate that decreased metabolic activity through nutrient deprivation may induce the formation of persister cells and represent a mechanism for increased antibiotic tolerance within polymicrobial cultures.

Interestingly, three articles from the Research Topic highlight how interactions between species can not only negatively affect the susceptibility of microbial populations to antimicrobials, but also be exploited for the identification of new drugs or drug targets. In the first of these articles, Hardy et al. screened a number of clinical bacterial isolates obtained from a variety of body sites for the ability to inhibit multiple *S. aureus* strains. They found that the majority of the isolates inhibited at least one *S. aureus* strain including MRSA. Furthermore, many of the clinical isolates belonging to the *Staphylococcus* and *Corynebacterium* genera mediated contact-independent inhibitory or bactericidal activity against *S. aureus* warranting the characterization of the active entities at the molecular level to reveal novel *S. aureus* therapeutics. In the second article, Herbrik et al. studied a strain of *Streptomyces* (TR1341) isolated from the sputum of a tuberculosis patient. They demonstrated that TR1341 produces at least two bioactive compounds with fungicidal or antibacterial/anti-virulence activity. Finally, in the third article (Veerapandian and Vediyappan), an *in vitro* study was carried out demonstrating the inhibition of mono-species or dual-species biofilms of *S. gordonii* and *C. albicans*, by gymnemic acid (GAs), a non-toxic small molecule inhibitor of fungal hyphae. The study shows that *S. gordonii* stimulates the expression of adhesive materials in *C. albicans* by direct interaction and/or signaling, and that the adhesive material expression can be inhibited by GAs.

Overall, we believe that the articles collected in this Research Topic represent a step forward for a better understanding of microbe-microbe interactions and their effects on infection outcome and antibiotic susceptibility. We hope that this article collection may encourage further studies in this research field aimed to develop new preventive and/or therapeutic approaches against poly-microbial infections.

## Author Contributions

GM and GB equally contributed to the writing of the manuscript. All authors contributed to the article and approved the submitted version.

## Conflict of Interest

The authors declare that the research was conducted in the absence of any commercial or financial relationships that could be construed as a potential conflict of interest.
